# Cardiomyocyte Na^+^/H^+^ Exchanger-1 Activity Is Reduced in Hypoxia

**DOI:** 10.3389/fcvm.2020.617038

**Published:** 2021-01-27

**Authors:** Hilmi Burak Kandilci, Mark A. Richards, Marjorie Fournier, Gül Şimşek, Yu Jin Chung, Samira Lakhal-Littleton, Pawel Swietach

**Affiliations:** ^1^Department of Physiology, Anatomy, and Genetics, University of Oxford, Oxford, United Kingdom; ^2^Department of Biophysics, Faculty of Medicine, Ankara University, Ankara, Turkey; ^3^Department of Biochemistry, University of Oxford, Oxford, United Kingdom

**Keywords:** NHE1, ventricle, oxygen, ATP, pH regulation, anemia, metabolism, Y557

## Abstract

Fully-activated Na^+^/H^+^ exchanger-1 (NHE1) generates the cardiomyocyte's largest trans-membrane extrusion of H^+^ ions for an equimolar influx of Na^+^ ions. This has the desirable effect of clearing excess intracellular acidity, but comes at a large energetic premium because the exchanged Na^+^ ions must ultimately be extruded by the sodium pump, a process that consumes the majority of the heart's non-contractile ATP. We hypothesize that the state of NHE1 activation depends on metabolic resources, which become limiting in periods of myocardial hypoxia. To test this functionally, NHE1 activity was measured in response to *in vitro* and *in vivo* hypoxic treatments. NHE1 flux was interrogated as a function of intracellular pH by fluorescence imaging of rodent ventricular myocytes loaded with pH-sensitive dyes BCECF or cSNARF1. Anoxic superfusates promptly inhibited NHE1, tracking the time-course of mitochondrial depolarization. Mass spectrometry of NHE1 immuno-precipitated from Langendorff-perfused anoxic hearts identified Tyr-581 dephosphorylation and Tyr-561 phosphorylation. The latter residue is part of the domain that interacts with phosphatidylinositol 4,5-bisphosphate (PIP_2_), a membrane lipid that becomes depleted under metabolic inhibition. Tyr-561 phosphorylation is expected to electrostatically weaken this activatory interaction. To test if a period of hypoxia produces a persistent inhibition of NHE1, measurements under normoxia were performed on myocytes that had been incubated in 2% O_2_ for 4 h. NHE1 activity remained inhibited, but the effect was ablated in the presence of Dasatinib, an inhibitor of Abl/Src-family tyrosine kinases. Chronic tissue hypoxia *in vivo*, attained in a mouse model of anemic hypoxia, also resulted in persistently slower NHE1. In summary, we show that NHE1 responds to oxygen, a physiologically-relevant metabolic regulator, ostensibly to divert ATP for contraction. We describe a novel mechanism of NHE1 inhibition that may be relevant in cardiac disorders featuring altered oxygen metabolism, such as myocardial ischemia and reperfusion injury.

## Introduction

Na^+^/H^+^ exchanger-1 (NHE1), a secondary active transporter coded by the *SLC9A1* gene (1), has an established role in regulating the intracellular pH (pH_i_) of cardiac myocytes (2, 3). When fully activated, NHE1 can produce the myocyte's largest transmembrane exchange of H^+^ and Na^+^ ions, allowing the rapid removal of excess acidity from cytoplasm (4). Whilst the NHE1 transport cycle does not hydrolyze ATP directly, it incurs an energetic cost because the imported Na^+^ ions must eventually be extruded by the Na^+^/K^+^ ATPase (“sodium pump”). Calculations performed for the beating heart suggest that two-thirds of cardiac ATP turnover is attributable to cross-bridge cycling (5, 6), and much of the remaining consumption is linked to primary active transport, of which the sodium pump accounts for half of the demand (7). Thus, around 10–20% of ATP turnover relates directly to Na^+^ transport, although this will vary depending on the state of the heart. In guinea-pig and rat beating hearts, the time-averaged flux carried by the sodium pump is 2–3 mM/min (8), which balances sarcolemmal Na^+^ influx carried by a number of pathways, including NHE1. However, maximal activation of NHE1 at low pH_i_ can evoke fluxes as large as >20 mM/min, which could deplete cellular ATP, particularly if the mitochondrial supply pipeline is restricted.

A key regulator of myocardial energetics is oxygen tension. In well-perfused myocardium, the high rate of oxidative metabolism in ventricular myocytes reduces blood oxygen saturation in the coronary sinus to 30% (9). Measurements of oxygen in the myocardium suggest partial pressures in the range 20–40 Torr, equivalent to 2.5–5% (10–12). However, oxygen levels will drop further toward anoxic levels when capillary flow is interrupted, for instance, in ischemia (10, 11, 13). We hypothesize that in periods of hypoxia, NHE1 activation is curtailed in order to ring-fence ATP for contraction. Thus, the aim of this study was to investigate how hypoxic treatments affect cardiac NHE1 activity in ventricular myocytes. Hypoxia was delivered *in vitro* by superfusion or incubation chamber, or *in vivo* by rendering animals anemic, which produces tissue hypoxia.

Early studies performed on non-cardiac cells demonstrated that ATP depletion drastically reduces the activity of NHE1 through a reduction in the transporter's apparent affinity for intracellular H^+^ ions (14, 15). This acute metabolic effect can take place without a change in NHE1 phosphorylation (16), and persists even when putative phosphorylation sites are eliminated by mutagenesis (16, 17). Instead, metabolic inhibition reduces NHE1 activity by depleting the membrane lipid phosphatidylinositol 4,5-bisphosphate (PIP_2_) (18). This weakens the activatory interaction between the plasma membrane and lipid-interacting domain (LID) at the NHE1 C-terminus. Lysine residues in the LID provide the positive charge that attracts NHE1 toward the membrane thereby activating ionic exchange. The strength of the membrane-LID interaction can also be modified hormonally by means of post-translational modifications to NHE1 (19).

We find that NHE1 activity is acutely inhibited by hypoxic conditions, and that some component of inhibition persists after more chronic episodes of hypoxia due to a post-translational modification to the LID. We conclude that NHE1 activity is highly responsive to metabolic status, which ensures that ionic exchange takes place only when metabolic resources are adequate.

## Materials and Methods

### Wild-Type Rat Ventricular Myocytes

Ventricular myocytes were isolated from rat hearts by enzymatic (protease and collagenase) and mechanical dispersion (20). Wistar rats (300–350 g) were sacrificed by stunning followed by cervical dislocation and confirmed by exsanguination in accordance with UK Home Office regulations (Schedule I of A(SP)A 1986), approved by national and University ethics committees. Hearts were rapidly removed and rinsed in isolation solution (in mM): 120 NaCl, 4 KCl, 1.2 MgCl_2_, 11 glucose, 10 HEPES, 2 NaH_2_PO_4_, 2.5 pyruvate, 20 taurine (MilliporeSigma) pH 7.4 at 37°C; supplemented with heparin (5 units/ml), and then mounted on a Langendorff perfusion system. Once cleared of blood, the heart was perfused with 1 mg/ml collagenase type II (Worthington) and 0.025 mg/ml protease type XIV (MilliporeSigma) for 10 min. Enzymatic activity was quenched by adding 1% BSA (MilliporeSigma) in isolation solution, cut into pieces, triturated with a Pasteur pipette and dissociated cells filtered through a 250-μm nylon mesh. Cells were washed once in 0.5 mM Ca^2+^ Tyrode and then once in 1 mM Ca^2+^ Tyrode prior to use.

### Mouse Model of Anemic Hypoxia

Animal procedures were performed in compliance with Home Office Guidance on the Operation of the Animals (Scientific Procedures) Act of 1986 and the University of Oxford institutional guidelines. All treatments administered to animals were approved by the Home Office under the Project License 30/3182, as described elsewhere (21). Male wild-type C57BL/6J mice used in this study were housed in individually ventilated cages, with a minimum of 2 and maximum of 6 mice per cage at the University of Oxford Biomedical Services Building. At 3 weeks of age, mice were weaned on either an iron-deficient diet (2–5 ppm iron; Teklad, TK99397; Envigo) or an iron-adjusted diet (200 ppm iron, Teklad, TK08713; Envigo). Hemoglobin concentration was measured from tail-vein blood, collected using a 27 G needle, using a HemoCue device (Radiometer). At 8 weeks of age, hearts were excise and cannulated at the aorta using a blunted 23-G needle, mounted on a Langendorff apparatus. The heart was first perfused with warm Tyrode perfusion solution containing (in mM): 130 NaCl, 5.6 KCl, 3.5 MgCl_2_, 5 HEPES, 0.4 Na_2_HPO_4_, 10 glucose, and 20 taurine (all from MilliporeSigma). The heart was then digested with Tyrode perfusion solution supplemented with 0.1 mM CaCl_2_ (MilliporeSigma), 1 mg/ml collagenase type II (Worthington) and 0.025 mg/ml protease (MilliporeSigma). Following digestion, cells were dissociated by careful mechanical disruption and filtered through a 500-μm cell strainer. Enzymatic activity was quenched by adding 1% BSA (MilliporeSigma) in Tyrode perfusion buffer. Cells were washed once in 0.5 mM Ca^2+^ Tyrode and then once in 1 mM Ca^2+^ Tyrode prior to use.

### Solutions and Superfusion

All chemicals were purchased from MilliporeSigma (UK) unless indicated otherwise. A superfusion system controlled the immediate environment of cells. Solutions were delivered at 37°C to a custom-made Perspex superfusion chamber with a coverslip glass bottom (for experiments on adult myocytes) or an Ibidi μ-slide (for experiments on cultured cells). Normal Tyrode contained (in mM) 135 NaCl, 4.5 KCl, 1 CaCl_2_, 1 MgCl_2_, 11 glucose, 20 HEPES at pH 7.4. In ammonium-containing solutions, NaCl was iso-osmotically replaced with NH_4_Cl. Calibration of cytoplasmic dye fluorescence as a function of pH_i_ was obtained using the K^+^/H^+^ ionophore nigericin (22). H^+^-efflux on NHE1 was calculated from the time-course of pH_i_ recovery in Hepes-buffered superfusates at pH 7.4, following a displacement of pH_i_ to an acidic level attained by a 4–6 min prepulse with 15 or 20 mM ammonium chloride. In some experiments 30 μM cariporide (a selective NHE inhibitor, Sanofi Aventis) was included in superfusates.

### Manipulating Oxygen Tension *ex vivo*

Anoxic solutions were prepared by vigorous bubbling with N_2_ gas in the presence of 1 mM sodium dithionite. Rapid and reversible anoxia was delivered to myocytes under superfusion by switching between normoxic and anoxic streams. Oxygen tension near myocytes was measured directly in the Perspex chamber using a needle type fiber optic oxygen probe (Microx Tx3, Presens). For longer incubation, cells we kept under an atmosphere of 2% O_2_ maintained by an Ibidi incubator (catalog no 11922). For parallel controls, cells were kept in a normal atmosphere of air. After incubation, NHE1 measurements were performed on cells under normoxic superfusion.

### Fluorescence Imaging

To measure intracellular pH, myocytes were AM-loaded with either cSNARF1 (10 μM, for 6 min) or BCECF (10 μM, for 10 min). cSNARF1 fluorescence from individual cells was measured using an inverted microscope (Nikon Diaphot, Telford, UK). cSNARF1 was excited with light (50 W xenon lamp) at 540 nm and the emitted fluorescence was measured simultaneously at 590 ± 10 nm and 640 ± 10 nm using two photomultiplier tubes linked to a current-voltage converter. The signals were then digitized at 0.5 kHz (CED 1401, Cambridge Electronic Design, Cambridge, UK). The digitized signals were integrated from both channels over 0.5 s and stored. The emission ratio (590/640 nm) was calculated and converted to a linear pH scale using the *in-situ* calibration data. Measurements with BCECF used a modification of the set-up that produced alternating excitation at 440 and 490 nm every 0.25 s, and fluorescence was collected at 535 ± 10 nm. JC-1 fluorescence were collected on a Zeiss confocal system using 488 nm laser excitation and collecting fluorescence ratiometrically at 530 and 600 nm, using a 570 nm dichroic mirror.

### Immunofluorescence Imaging

Freshly isolated primary cardiomyocytes were allowed to adhere to laminin-coated Ibidi μ-slides (Ibidi, 81201) prior to being fixed with 4% PFA for 15 min and permeabilised using 0.5% Triton X-100 for 4 min at room temperature (RT). Non-specific antibody binding was blocked by treating myocytes with blocking solution of 5% BSA in PBS with 0.1% Triton X-100 for 1 h at RT. Primary antibody raised against NHE1 (Millipore, AB3081) at 1:50 diluted in blocking solution was applied to myocytes overnight at 4°C. Donkey anti-rabbit Alexa Fluor® 647 secondary antibodies (Abcam, ab150075) were diluted to 1:1,000 in blocking solution and incubated with myocytes for 1 h at RT. Coverslips were mounted onto μ-slides with ProLongTM Diamond mounting media including the nuclear stain DAPI (Thermo). Images were captured by confocal microscopy using a Leica SP5 system.

### Immunoprecipitation

Adult rat hearts were removed and retrogradely perfused using the Langendorff perfusion technique with either normoxic or anoxic (100% N_2_ with 1 mM sodium dithionite) Normal Tyrode's solution for 15 min. Ventricular tissue was then dissected and snap frozen in liquid N_2_. Protein samples were obtained by homogenization of ventricular tissue in ice cold lysis buffer containing protease and phosphatase inhibitors (Roche). Insoluble cellular components were removed by centrifuging samples at 12,000 rpm at 4°C for 20 min, supernatants were retained for immunoprecipitation. To immunoprecipitate NHE1, 250μg of each protein sample was incubated with 2 μg of anti-NHE1 antibody (Abnova, H00006548-M01) at 4°C overnight. Antibody-NHE1 complexes were mixed with Protein A/G Agarose beads (Thermo, 20421) for 2 h at room temperature to bind the antibody-NHE1 complexes. Beads were collected using centrifugation by retaining the pellet and discarding the supernatant containing unbound protein. Immunoprecipitated NHE1 was eluted from the beads by adding Laemlli buffer containing beta-mercaptoethanol to the pellet and heating at 65°C for 3 min. Eluted NHE1 samples were electrophoresed in a 10% SDS-PAGE gel then visualized using coomassie stain prior to being excised from the gel and stored at −20°C for analysis by mass spectrometry.

### Mass Spectrometry-Based Proteomics

LC-MS/MS: peptides were resuspended into 5% formic acid and 5% DMSO and separated by nano liquid chromatography (Easy nLC 1000) coupled in line a Q Exactive mass spectrometer equipped with an Easy-Spray source (Thermo Fischer Scientific). Peptides were trapped onto a C18 PepMac100 precolumn (300 μm i.d. × 5 mm, 100 Å, ThermoFischer Scientific) using Solvent A (0.1% Formic acid, HPLC grade water). The peptides were further separated onto an Easy-Spray RSLC C18 column (75 μm i.d., 50 cm length, Thermo Fischer Scientific) using linear gradients of either 15 or 60 min, at a flow rate 200 nl/min. The raw data were acquired on the mass spectrometer in a data-dependent acquisition mode (DDA). Full-scan MS spectra were acquired in the Orbitrap (Scan range 350–1,500 m/z, resolution 70,000; AGC target, 3e6, maximum injection time, 50 or 100 ms). After the MS scans, either 5 or 10 most intense ions were selected for higher-energy collision dissociation (HCD). HCD spectra were acquired in the Orbitrap (resolution,17.500; AGC target 5e4; maximum injection time, 120 ms) with fixed mass at 180 m/z. Data analysis: tandem mass spectra were searched using SEQUEST HT within Proteome discoverer PD1.4 (Thermo Fischer Scientific, version 1.4.0.288) against the NHE1/SL9A1 protein sequence from *Rattus norgevicus* (uniprot ID, P26431). During database searches, cysteines (C) were considered to be fully carbamidomethylated (+57.0215, statically added), methionine (M) to be fully oxidized (+15.9949, dynamically added), N-terminal lysine (K) to be acetylated (+42.0106, dynamically added), and serine (S), threonine (T) and tyrosine (Y) to be phosphorylated (+79.9963, dynamically added). Two missed cleavages were permitted. Peptide mass tolerance was set at 50 ppm and 0.02 Da on the precursor and fragment ions, respectively. The protein identification was filtered at FDR below 1%. Phosphorylated tandem mass spectra were manually validated. Data are available via ProteomeXchange with identifier PXD022853.

### Statistics

Summary data are reported as mean ± SEM. Number of observations are reported as “number of cells, number of animals.” Nested analysis was performed to test for clustering of data by isolation. Two-way ANOVA tested for a significant effect of hypoxia and interaction with pH (i.e., a change in pH-sensitivity) in pH_i_-NHE1 flux datasets between a hypoxic group and its control. NHE1 measurements were performed on myocytes isolated from at least three animals. To study the acute effects of anoxia/hypoxia, measurements were performed on cells that were superfused with anoxia/hypoxia, or normoxic solution. The treatment decision was randomized and ensured that each cell isolation yield included control and treatment groups. To study the effects of 4 h hypoxic incubation, a yield of cells was split into two vessels and placed in a hypoxic incubator or in air. Each yield of cells therefore included control and treatment groups. For *in vivo* hypoxia, mice were randomized to iron-deficient or normal diet, and cells obtained after 5 weeks of dietary intervention. Thus, any one heart would yield either control or hypoxia-exposed myocytes. For other types of statistical testing, two-sided *t*-tests were performed. In figures, one asterisk (*) denotes a *P* value between 0.01 and 0.05, and two asterisks (**) denote a *P* value smaller than 0.01.

## Results

### Cardiac NHE1 Activity Is Rapidly and Profoundly Inhibited by Acute Hypoxia

**Figure 1:**
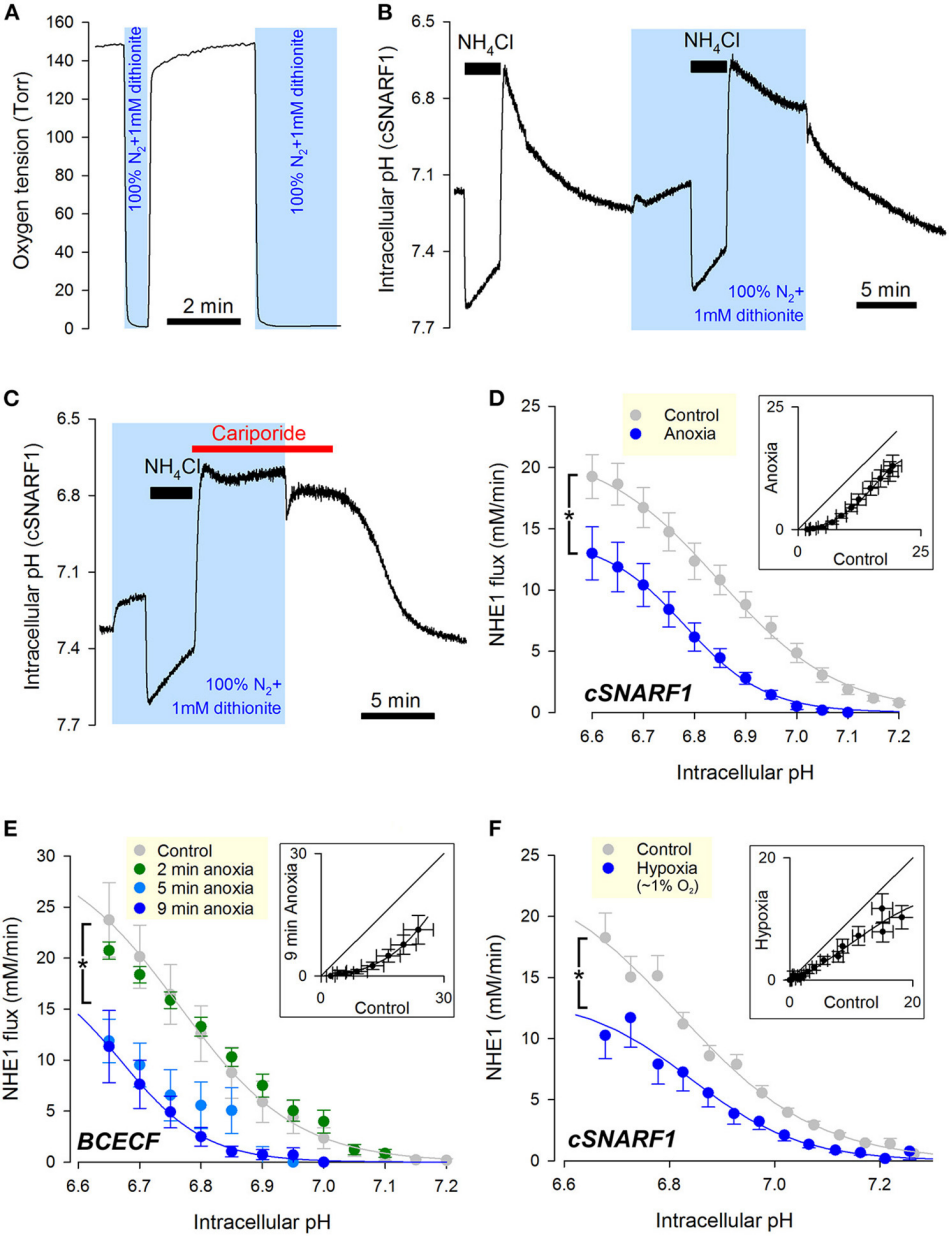
Cardiac NHE1 activity is rapidly inhibited by acute anoxia. **(A)** Measurement of oxygen tension in the superfusion chamber, showing anoxic conditions are attainable by solutions bubbled with N_2_ and supplemented with the oxygen-scavenger dithionite (1 mM). **(B)** Exemplar trace of paired ammonium prepulses, first performed in normoxia and then in anoxia, in a rat ventricular myocyte loaded with cSNARF1. Recovery of pH_i_ from the acid load is greatly attenuated under anoxic conditions, but becomes re-activated upon re-oxygenation. **(C)** Exemplar time course showing lack of background acid-loading at low pH_i_ under anoxic conditions, revealed in the presence of cariporide (30 μM) to block NHE1. **(D)** NHE1-generated H^+^-flux measured by cSNARF1 or **(E)** BCECF in normoxia (cSNARF1: *n* = 22 cells from 5 rats; BCECF: *n* = 9 cells from 4 rats) or 9 min anoxia (cSNARF: *n* = 9 cells from 2 rats; BCECF: *n* = 7 cells from 2 rats). In the case of BCECF experiments, the effect of anoxia was also tested after 2 min exposure (*n* = 5 cells from 3 rats), 5 min exposure to anoxia (*n* = 5 cells from 3 rats). Two-way ANOVA: effect of 9 min anoxia vs. control was significant (*P* < 0.0001 for cSNARF1 and *P* < 0.01 for BCECF datasets); effect of 5 min anoxia vs. control was significant (*P* < 0.0001); effect of 2 min anoxia was not significant (*P* = 0.93). Interaction between pH and treatment was not significant in any of the cases; the effect of pH_i_ was significant (*P* < 0.0001 for all datasets). Insets plot flux in anoxia (9 min) vs. normoxia for matching pH_i_; the parallel shift is indicative of a change in affinity. **(F)** Effect of 15 min 3 Torr hypoxia (~1% O_2_) attained by including 0.1 mM dithionite in N_2_-bubbled solutions. Data recorded with cSNARF1. NHE1 activity was inhibited by hypoxia (*n* = 26 cells, 3 rats) relative to controls (*n* = 55, 3 rats). Two-way ANOVA: significant effect of hypoxia (*P* < 0.001); of pH_i_ (*P* < 0.0001); and interaction (*P* < 0.0001). Inset plots flux in anoxia vs. normoxia for matching pHi. The observed parallel shift is indicative of a change in affinity.

The first hypoxic treatment performed on myocytes to test for NHE1 responses was acute anoxia. To manipulate oxygen tension, superfusion was switched between a “normoxic” stream (pre-equilibrated with 21% O_2_; 160 Torr) and an “anoxic” stream (0 Torr; produced by bubbling solution with 100% N_2_ and supplementing with the oxygen scavenger dithionite, 1 mM). Manual switching between these solutions changed the immediate milieu of cardiac myocytes within seconds (Figure 1A). Cardiac NHE1 was interrogated functionally from measurements of pH_i_ in enzymatically isolated rat ventricular myocytes loaded with a pH-sensitive indicator. NHE1 activity was triggered by means of an ammonium prepulse solution maneuver, which acid-loads the cytoplasm typically down to pH ~6.6. NHE1 transport activity is independent of CO_2_/${\text{HCO}}_{3}^{-}$ buffer, and was therefore studied under a buffering regime consisting of 20 mM HEPES buffer. An exemplar recording (Figure 1B) shows pH_i_ recovery in normoxia followed by a recovery in anoxia (shaded), measured using the pH-indicator dye cSNARF1. Note that upon applying anoxia, the myocyte responded with a slow acidification due to background acid-loading. Recovery of pH_i_ was markedly slower in anoxia, but resumed promptly upon re-oxygenation. The pH_i_ recovery in anoxia was attributable to NHE1, as it was wholly inhibited by NHE1-selective blocker cariporide (30 μM). This result also indicates that a period in anoxia does not activate background acid-loading at low pH_i_, which would oppose NHE1 and slow the pace of recovery (Figure 1C).

**Figure 2:**
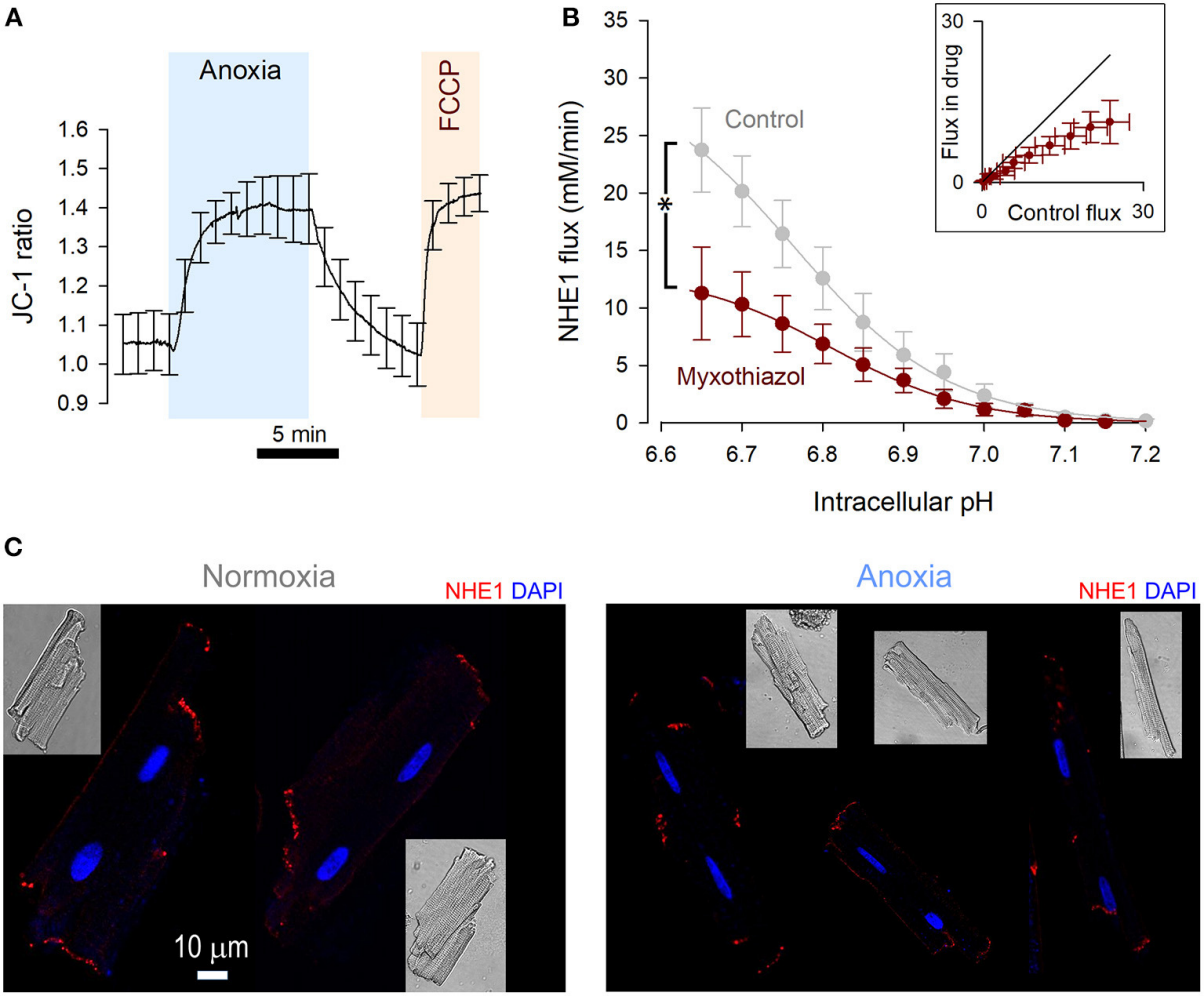
NHE1 activity is reduced by metabolic inhibition. **(A)** JC-1 fluorescence ratio measured in rat ventricular myocytes (*n* = 12 cells from 4 rats), showing mitochondrial depolarization in response to anoxic conditions. The response was comparable to the effect of FCCP (5 μM), indicating a complete depolarization by anoxia. **(B)** NHE1-generated flux measured in ventricular myocytes in the presence myxothiazol (1 μM, *n* = 7 cells from 3 rats), compared to controls. Two-way ANOVA: significant effect of drug (*P* < 0.0001); of pH_i_ (*P* < 0.0001); and interaction (*P* = 0.0461). Inset plots flux in the presence and absence of inhibitor for matching pHi. **(C)** Immunofluorescence of rat ventricular myocytes for NHE1 under control conditions and after treatment with anoxic conditions for 15 min. NHE1 distribution appears unaltered. Scale bar applies to both images.

To quantify the effect of anoxia on NHE1, a cell under superfusion was randomized to receive anoxia for 5 minutes prior to NHE1 activation, or remain in normoxic conditions (control). To calculate the NHE1-generated flux, the rate of pH_i_-change was multiplied by buffering capacity (β), measured in separate experiments using a protocol consisting of step-wise reductions in ammonium (intrinsic buffering was unaffected by acute anoxia; Supplementary Figure 1). Fluorescence recordings were performed using either cSNARF1 (Figure 1D) or BCECF (Figure 1E) to eliminate dye-dependent responses to anoxia, e.g. possible chemical changes to dye. A plot of NHE1-generated transmembrane H^+^ flux against pH_i_ revealed a left-shift under anoxic conditions, irrespective of the dye used (Figures 1D,E). The inhibitory effect can be visualized by plotting anoxic flux vs. normoxic flux at matching pH_i_ (inset), revealing a relationship that is shifted parallel to the identity line. Such a kinetic response is characteristic of a change in apparent affinity, rather than maximal transporter turnover (V_max_). To measure the rate of onset of the anoxic effect on NHE1, oxygen tension was reduced to anoxic levels at 2, 5, or 9 min prior to NHE1 activation. Two minutes of anoxia was not sufficient to reduce NHE1 activity, but a profound inhibition became apparent after at least 5 min of anoxic treatment (Figure 2E). Of the two dyes used, cSNARF1 was mildly sensitive to an anoxic chemical environment. When pH_i_ was clamped to extracellular pH by the protonophore nigericin, anoxia evoked a small but significant change in cSNARF1 fluorescence ratio (Supplementary Figure 2). The magnitude of this effect is, however, too small to explain the oxygen-sensitivity of NHE1.

An additional set of experiments was performed on myocytes that had been exposed to a milder form of hypoxia (3 Torr) attained with a lower (0.1 mM) concentration of dithionite delivered 15 min before NHE1 activation, and compared to normoxic controls paired from the same cell isolation. The milder hypoxic treatment, which is below the physiological O_2_ tension of the beating myocardium *in vivo*, also reduced NHE1 activity, with the characteristic left-shift in the activation curve (Figure 1F), but the effect was less pronounced than that with anoxia, indicating a dose-dependence between oxygen levels and NHE1 activity.

### The Action of Anoxia on NHE1 Tracks the Response of Mitochondrial Respiration

The ATP-sensing function of NHE1 is the putative mechanism for the acute inhibition in anoxia, which required 5 min to fully engage. To determine the rate and extent to which anoxia affects mitochondrial respiration, mitochondrial membrane potential was measured using the ratiometric dye JC-1 loaded into myocytes (Figure 2A). As a positive control for mitochondrial depolarization, FCCP (5 μM) was added at the end of the protocol. The change in JC-1 ratio was quantitatively similar to that evoked by FCCP, indicating that the anoxic milieu produces a robust inhibition of oxidative phosphorylation. This inhibitory effect required 5 min to complete, which matches the time course of NHE1 inhibition by a similar treatment regime.

If mitochondrial respiration were critical for enabling NHE1 activity, then the pharmacological inhibition of electron transport chain complexes should also reduce sarcolemmal NHE1 activity. Indeed, myocytes treated for 9 min with 1 μM myxothiazol, inhibitor of Complex III, produced smaller NHE1 fluxes (Figure 2B). Inhibition of mitochondrial respiration thus produces a rapid effect on NHE1, akin to anoxia.

A previous study linked energy status with NHE1 internalization (23), yet immunofluorescence imaging of Triton-X permeabilized myocytes showed no clear effect of anoxia on the distribution of NHE1, which remained associated with the intercalated disc region (Figure 2C) (24). Given the effect-size of anoxia on NHE1-generated flux, internalization of protein is unlikely to be the main mechanism of action, but a contributing role for it cannot be excluded.

### Post-translational Changes to NHE1 Under Anoxic Conditions

**Figure 3:**
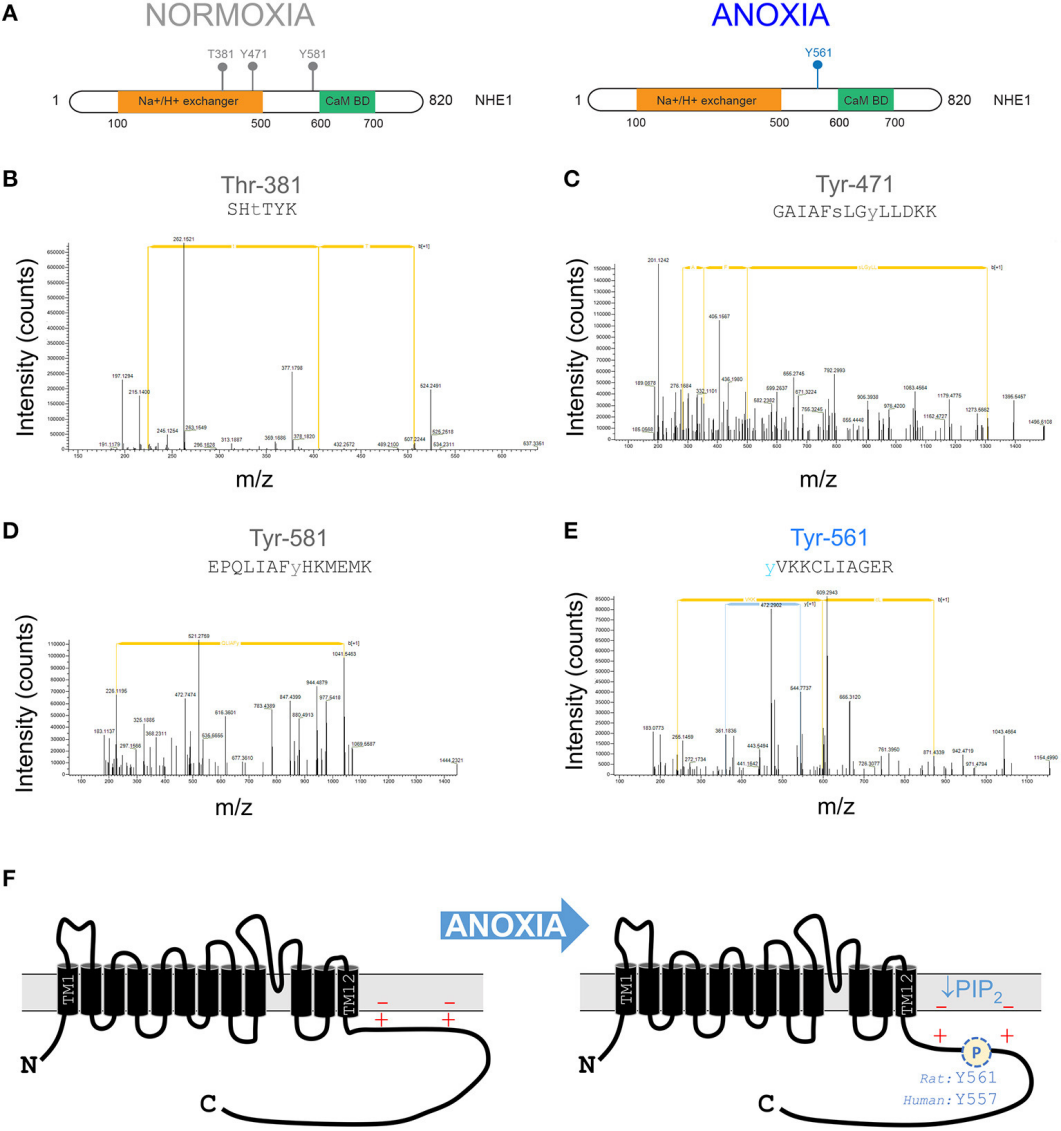
Anoxia changes the phosphorylation pattern at the NHE1 C-terminus. **(A)** Phosphorylation sites identified by tandem mass spectrometry-based proteomics analysis on NHE1 pulled-down from rat hearts Langendorff-perfused with either anoxic (*n* = 4) or normoxic (*n* = 4; control) buffers for 15 min prior to sample collection. Threonine residue at position 381 (T381) and tyrosine residues at positions 471 (Y471) and 581 (Y581) were identified in normoxia conditions, while tyrosine residue at position 561 (Y561) was identified in hypoxia conditions only. The Na^+^/H^+^ exchanger region and calmodulin binding domain (Cam BD) are highlighted in orange and green, respectively, on NHE1 protein. **(B–D)** Tandem mass spectra containing phosphorylated T381, Y471, Y581, and Y561 residues matching the corresponding peptide ions in **(B)** SHTTIK (*z* = +2, *m*/*z* = 383.68954), **(C)** GAIAFSLGYLLDKK (*z* = +2, *m*/*z* = 849.36743), **(D)** EPQLIAFYHKMEMK (*z* = +3, *m*/*z* = 615.64807) and **(E)** YVKKCLIAGER (*z* = +3, *m*/*z* = 472.89618) are shown. **(F)** Schematic of NHE1 protein structure, showing positively charged lipid interacting domain of the C-terminus. Anoxia leads to phosphorylation which weakens the otherwise activatory interaction with negatively charged PIP_2_ in the inner leaf of the sarcolemma. Additionally, metabolic inhibition will deplete PIP_2_ levels.

After an initial period of metabolic inhibition, anoxia may evoke additional changes to the NHE1 protein that influence its activity. Indeed, NHE1 is subject to a myriad of post-translational modifications (PTMs), including several phosphorylation sites in the C-terminus. To identify oxygen-sensitive changes in NHE1 phosphorylation, mass spectrometry-based proteomics analysis was performed on samples of rat ventricles. Hearts were Langendorff-perfused with anoxic medium (N_2_-bubbled, containing 1 mM dithionite) for 15 min, followed by snap freezing in liquid nitrogen. For controls, hearts were perfused with normoxic medium. NHE1 protein was immunoprecipitated from samples of left ventricle. In total, four “normoxic” and “anoxic” hearts were processed for mass spectrometric analysis to seek PTMs. At a false-discovery rate better than 1%, four phosphosites were identified as significantly different between the two groups. Following manual validation, the identified phosphosites were phosphorylated at Thr-381, Tyr-471, and Tyr-581 in normoxia, and phosphorylated at Tyr-561 in anoxia (Figures 3A–E). Tyr-581 and Tyr-561 (equivalent to Tyr-577 and Tyr-557 in the human NHE1 protein) are part of the C-terminus, which is known for its role in regulating ion exchange (Figure 3F). Anoxia-evoked Tyr-561 phosphorylation is significant for the LID because it introduces a negative charge among lysine residues, predicted to weaken the electrostatic interaction with membrane-anchored PIP_2_. Such an effect would synergize with the inhibitory effect of PIP_2_ depletion, possibly producing a longer-lasting effect of hypoxia on NHE1.

### Cardiac NHE1 Activity Is Inhibited Persistently After Hypoxic Incubation

A post-translational modification to NHE1 may produce more persistent effects on activity, that may remain even after normal O_2_ tension is restored. To investigate this, a yield of cells was split into two dishes, with one incubated for 4 h in an atmosphere of 2% oxygen and the other time-matched in normal air as the control. This duration is sufficient to stabilize any NHE1 post-translational modifications and metabolic remodeling, without meaningful changes in gene expression that would normally require overnight treatment. 2% O_2_ is lower than physiological O_2_ in the beating myocardium, and thus represents a hypoxic state. Lower tensions were found to cause excessive cell death over 4 h and therefore not selected for this experiment.

**Figure 4:**
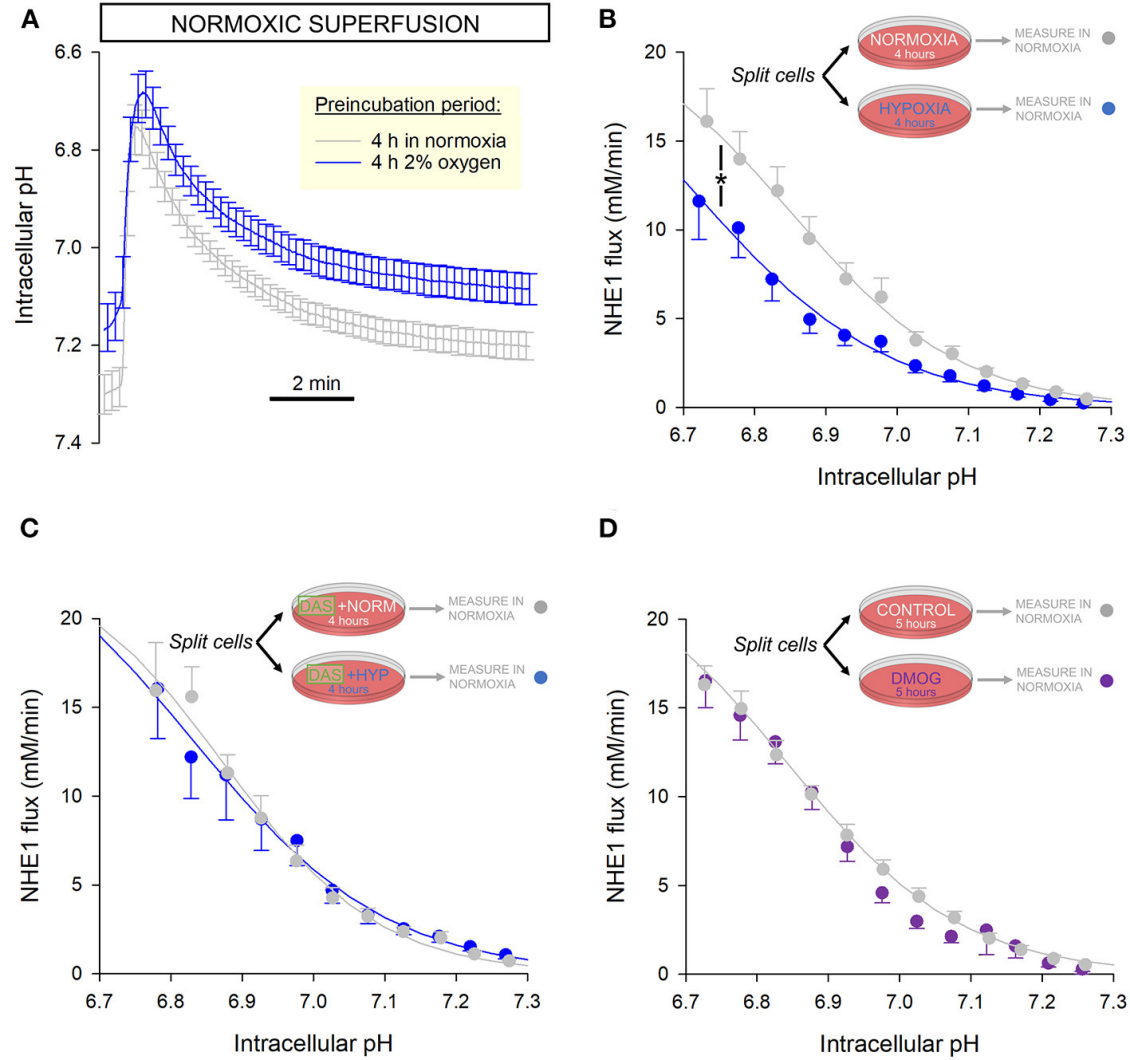
NHE1 activity is persistently inhibited following 4 h incubation in hypoxia. **(A)** Freshly isolated rat myocytes were split and incubated either in an atmosphere of 2% O_2_ or normal air. Low O_2_ was maintained by under an Ibidi Chamber. After incubation, NHE1 activity was measured in myocytes under normoxic superfusion, within 30 min of re-oxygenation (*n* = 30 control cells and 28 hypoxia-pretreated cells from 4 rats). **(B)** NHE1 activity remained lower in cells that had been exposed to hypoxia, indicating that the inhibitory effect of low O_2_ persists after re-oxygenation. Two-way ANOVA: significant effect of treatment (hypoxic pre-incubation) (*P* < 0.0001) and interaction between treatment and pH (*P* < 0.0001). **(C)** Experiments were repeated in the presence of 100 nM Dasatinib, a Abl/Src tyrosine kinase inhibitor. In the presence of inhibitor, hypoxia had no effect on NHE1, consistent with the role of Y561 phosphorylation (*n* = 15 controls cells, 16 hypoxia pre-treated cells; from 3 rats); two-way ANOVA: no significant effect of treatment (hypoxic pre-incubation) in presence of Dasatinib (DAS) (*P* = 0.96); no significant interaction with pH (*P* = 0.98). **(D)** 5 h treatment with 1 mM DMOG had no effect on NHE1 in rat myocytes, compared to controls (*n* = 34 controls cells, 27 DMOG-treated cells from 4 animals); two-way ANOVA: no significant effect of treatment (DMOG) (*P* = 0.78).

After incubation, NHE1 activity was measured in cells superfused with normoxic solutions to determine whether NHE1 inhibition is manifested even after re-oxygenation. These assays were performed within 30 min of re-oxygenation. The pH_i_ recovery time courses were slower in hypoxia pre-incubated myocytes, compared to their time-matched controls (Figure 4A). The reduction in NHE1 activity indicates a more persistent legacy of the hypoxic period (Figure 4B).

Based on proteomics analyses, the putative mechanism of NHE1 inhibition may relate to hypoxia-evoked Tyr-561 phosphorylation. This may be catalyzed by c-Src or c-Abl tyrosine kinases (25), as these enzymes act on a consensus sequence that is related to the motif containing Tyr-561 (KKYVK). To test this, experiments were repeated in the presence of 100 nM Dasatinib, a membrane-permeant, small-molecule inhibitor of Abl and Src family tyrosine kinases. A yield of cells was split into two groups, one of which was incubated in 2% O_2_ with Dasatinib, and the other (its control) in normal air with Dasatinib. After a 4 h incubation period, NHE1 activity was measured in normoxic superfusates, without Dasatinib. In these experiments, prior hypoxia had no inhibitory effect on NHE1, indicating the involvement of Abl/Src kinases in the inhibitory actions of low O_2_ tension (Figure 4C). This finding is consistent with the role of Tyr-561 in transducing an oxygen signal onto NHE1.

During 4-h hypoxic incubation, it is plausible that prolyl hydroxylase (PHD) enzymes become inhibited, causing a change in protein hydroxylation. As a post-translational modification, proline hydroxylation may meaningfully affect the function of proteins. Although NHE1 lacks the consensus LxxLAP motif for PHD binding, the regulatory cytoplasmic C-terminus has 26 proline residues that could potentially be hydroxylated. To test if hypoxia may be acting on NHE1 via a PHD-dependent change in hydroxylation, myocytes were incubated for 5 h with the PHD inhibitor dimethyl oxalylglycine (DMOG; 1 mM), followed by measurements in DMOG-free superfusates. NHE1 measured in DMOG-pretreated rat myocytes was not significantly different to time-matched controls without DMOG, indicating that NHE1 activity is not regulated by changes in hydroxylation (Figure 4D).

### Cardiac NHE1 Activity Is Persistently Reduced in Hypoxic Myocardium *in vivo*

We have shown that an *in vitro* hypoxic treatment lasting several hours produces a persistent inhibition of NHE1. Due to limitations in cell culture, it is not possible to test for the effect of more chronic tissue hypoxia, which may, for example, result from a sustained inadequacy of myocardial perfusion. The effects of chronic hypoxia were therefore determined in a mouse model of anemia, which has impaired convective O_2_ transport and produces markers of cardiac hypoxia, including the stabilization of HIF-1 and upregulation of its target gene (21). Mice were rendered anemic by weaning on an iron-deficient diet (2–5 ppm iron) for 5 weeks so that by 8 weeks of age, hemoglobin levels fell to 50–60 g/L (relative to 130–145 g/L in animals on a normal diet of 200 ppm iron). The iron-deficient diet was found to produce a more robust and consistent reduction in blood hemoglobin levels in mice compared to rats. Hearts of anemic mice underwent a shift toward a glycolytic phenotype, which impairs cardiac energetics (26), which, according to the proposed model, should also affect NHE1 activity.

**Figure 5:**
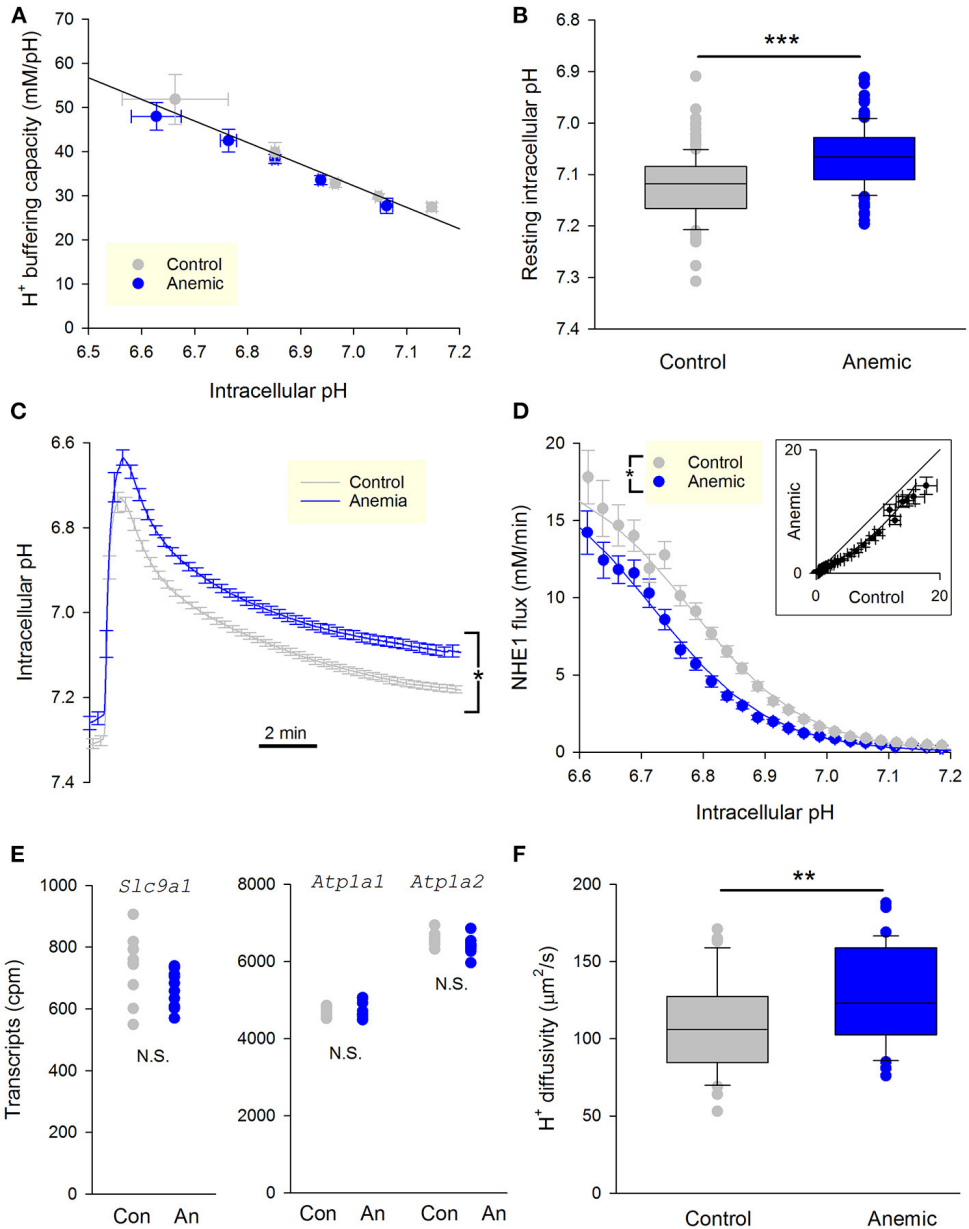
Chronic myocardial hypoxia in anemic animals persistently reduces NHE1 activity. **(A)** Intrinsic buffering capacity in myocytes from control (*n* = 20 cells, 3 mice) and anemic mice (*n* = 23 cells, 3 mice). No significant difference (two-way ANOVA). **(B)** Resting pH_i_ under Hepes-buffering regime (*n* = 124 and 121 cells from 3 anemic and 3 control mice, respectively); *t*-test, *P* < 0.01. **(C)** Time course of pH_i_ recovery following an ammonium prepulse. Average of 46 myocytes from three control hearts and 36 myocytes from three anemic hearts. Error bars not shown for clarity. **(D)** NHE1 generated flux in myocytes from control (*n* = 46 cells from 3 mice) and anemic (*n* = 36 cells from 3 mice) animals, showing significant decrease in the latter; two-way ANOVA: significant (*P* < 0.01) effect of anemic intervention. **(E)** Levels of transcripts for the gene coding NHE1, *Slc9a1*, is not different between anemic and control hearts. Whole heart tissue mRNA from 9 mice per group. Used as a control, Na^+^/K^+^ pump genes *Atp1a1* and *Atp1a2* are unchanged. **(F)** Apparent H^+^ diffusion coefficient is significantly increased in myocytes from anemic mice (*n* = 12 from 3 mice) relative to control myocytes (*n* = 12 from 3 mice); *t*-test, *P* < 0.01.

Enzymatically-isolated ventricular myocytes were loaded with the pH-sensitive dye cSNARF1, and imaged within 3 h of isolation. Buffering capacity was no different between myocytes from anemic and control mice (Figure 5A). In contrast, resting pH_i_ was significantly reduced in myocytes from anemic mice (Figure 5B). NHE1 flux was measured by ammonium prepulse. The time course of pH_i_ recovery was markedly slower in myocytes from anemic mice (Figure 5C), indicative of slower NHE1 activity and consistent with the more acidic resting pH_i_. Myocytes isolated from a hypoxic myocardium manifested a left-shifted activation curve, which indicates NHE1 inhibition (Figure 5D). The pH_i_-flux curves could be fitted to Hill-type curves to estimate maximum flux (V_max_), cooperativity, and pH at which activity is half-maximal (pK_a_). The effect of chronic hypoxia is best described by a decrease in pK_a_. Notably, tissue hypoxia did not affect V_max_, which argues against a change in the total number of NHE1 molecules at the cell surface. In support of this, analysis of RNA-sequencing data for mice from this cohort of experiments showed no difference in *Slc9a1* transcript levels, the gene coding for NHE1 (Figure 5E).

For NHE1 to produce a transmembrane flux, it has to be adequately supplied with cytoplasmic H^+^ ions (27). This supply pipeline requires mobile H^+^ buffers, as the high buffering capacity of cytoplasm greatly restricts free H^+^ ion movement. Apparently lower NHE1 activity in myocytes from hypoxic myocardium could arise from reduced levels of mobile buffers. To test this, the apparent H^+^ diffusion coefficient was measured by imaging the diffusive spread of H^+^ ions from a point source of acid maintained by a series of photolytic uncaging events limited to one end of the cell only, as described previously (27). The photolabile H^+^ donor was 2-nitroveraldehyde, included at 1 mM in solutions (28). Intriguingly, H^+^ diffusivity increased modestly in myocytes from the anemic mouse (Figure 5F). This observation indicates that the decrease in NHE1 flux cannot be due to impaired diffusive H^+^ coupling across the cytoplasm. In summary, the shift from mitochondrial to glycolytic metabolism in chronically-hypoxic ventricular myocytes produces a stable decrease in NHE1 activity.

## Discussion

The results of this study demonstrate a functional coupling between oxygen tension and the ionic flux carried by NHE1 in the heart. A sudden decrease in oxygen availability produces a prompt fall in NHE1 activity, manifested by a left-shift in the pH-activation curve (i.e., in the acidic direction). This NHE1 response tracks the response of mitochondrial membrane potential, arguing that it is secondary to metabolic depletion. NHE1 inhibition was more profound under anoxia compared to ~1% O_2_, indicating a dose-dependence of O_2_-NHE1 coupling, putatively in proportion to the extent of metabolic depletion. In this respect, cardiac NHE1 responds in a similar way to the non-cardiac protein. The most widely accepted mechanism for the acute effect of metabolic depletion on NHE1 implicates PIP_2_ depletion and the resulting weakening of an activatory interaction between the NHE1 C-terminus and membrane (18). Alternative models have proposed a role for direct ATP binding to NHE1 (29) and cannot be discounted. NHE1 internalization in response to hypoxia (30) is unlikely to be the dominant mechanism in primary cardiac cells, based on immunofluorescence evidence and kinetic analyses that indicate a shift in H^+^ affinity, rather than fall in maximal flux (V_max_).

Proteomic analyses of anoxia-treated cardiac NHE1 highlight a novel post-translational modification at Tyr-561, a residue that is adjacent to several lysine residues interacting with membrane-anchored PIP_2_, and part of the consensus sequence for Abl/Src tyrosine kinases. Phosphorylation of this tyrosine (equivalent to Tyr-557 in the human protein) would weaken the interaction between the C-terminus and the membrane, and therefore synergize with PIP_2_ depletion to reduce NHE1 activity. The effect of this post-translational modification persists for at least ~30 min after re-oxygenation, as shown by the persistent NHE1 inhibition in myocytes incubated under hypoxia for 4 h *in vitro*. This effect was not mimicked by 5 h treatment of cells with DMOG, an inhibitor of prolyl hydroxylases (31, 32), arguing against a role of hydroxylation. Prolonged hypoxic incubation will produce metabolic changes in the cardiomyocyte, such as a gradual depletion of intracellular ATP (33), but these are likely to be restored promptly upon reoxygenation. A 4 h period of hypoxia is unlikely to change the expression of proteins coded by hypoxia-sensitive genes, although some changes in gene expression may occur with longer hypoxic episodes (34). Strikingly, the persistent component of NHE1 inhibition was absent when Abl/Src tyrosine kinases were inhibited pharmacologically with Dasatinib. These findings describe the role of a hitherto uncharacterized phosphorylation site in the regulation of NHE1. Abl/Src kinases are attractive candidates for oxygen sensing as they are known to become activate in hypoxia (35, 36) and under oxidative stress (37–39). Inhibition of Na^+^ influx is also consistent with the pro-survival actions of Src kinases observed in cardiomyocytes (40).

The NHE1 response to more chronic hypoxia cannot be studied *in vitro* due to poor survival of primary myocytes, but the effect could be inferred from an animal model of tissue hypoxia. A way of achieving sustained tissue hypoxia is by inducing anemia in animals feed an iron-deficient diet. The hearts from iron-deficient animals exhibit signs of hypoxia, such as up-regulation of the HIF-α protein and induction of its target genes (21, 26). Although global and complex transcriptomic alterations are observed in the hearts of anemic mice, these effects have been attributed largely to anemic hypoxia rather than iron deficiency (26). Importantly, metabolism switches to a more glycolytic phenotype in the anemic myocardium compared to controls, an effect which is characteristic of hypoxia (26). Cardiac myocytes from anemic animals had lower NHE1 activity, with unaltered expression of its gene (*Slc9a1*) nor V_max_. In summary, *in vitro* and *in vivo* models presented here demonstrate an inhibitory effect of hypoxia on NHE1 activity.

In the anoxic state, NHE1 activity was reduced substantially, producing the equivalent of a 0.2 pH-unit leftward shift in the activation curve. In units of [H^+^], anoxia elevates the threshold for activating NHE1 by ~50%. Whilst such an action dampens the regulatory prowess of pH_i_ control, it offers a substantial saving in terms of ATP because Na^+^-influx pathways tap directly to the myocyte's energy pool. Given that resting Na^+^ entry (2–3 mM/min) can consume 10–20% of ATP produced by the myocyte, a full activation of NHE1 (>20 mM/min) could consume the majority of ATP for as long as the acid-challenge persists. When mitochondrial respiration is reduced under hypoxic conditions, ATP turnover is severely affected, and in order to allocate sufficient resources to contraction, other ATP-consuming processes need to be turned down. The hypoxia-responsiveness of NHE1 activity is thus an example of an austerity measure that comes at the cost of diminished protection against acidic disturbances. We interpret this response to indicate that the heart prioritizes ATP for contraction at the cost of housekeeping functions, such as pH_i_ control, even though these will eventually influence contraction.

From a methodological viewpoint, this study demonstrates that superfusion can exercise rapid control over the oxygenation status of myocytes, and produce anoxic conditions when solutions are bubbled with N_2_ and supplemented with dithionite. Our work also highlights an advantage of imaging with BCECF over cSNARF1, as the former showed no sensitivity to anoxic conditions, whereas measurements with the latter entailed a small yet significant artifact, ostensibly due to the redox chemistry of the dye.

The significance of this study is that a major Na^+^-influx pathway through NHE1 can be titrated in accordance with metabolic state. Although NHE1 is typically presented as a robust regulator of pH_i_, our study argues that it is a relatively labile component, highly sensitive to the state of cellular signaling. Such plasticity may be necessary for NHE1 to remain responsive to dynamic changes in the beating myocardium. In ischemia, the hypoxic effect will synergize with the inhibitory action of extracellular acidosis to produce a robust decrease in NHE1 activity. Rapid re-oxygenation upon reperfusion will eventually accelerate NHE1 flux, but the recovery may be slowed by a component of “hypoxic” memory in the form of Tyr-561 phosphorylation, a novel inhibitory post-translational modification that persists for at least 30 min after re-oxygenation. This modification may protect from excessive damage as part of ischemia-reperfusion injury, in which Na^+^ influx has been linked to arrhythmias (41, 42). At the other extreme, a well-oxygenated myocardium is expected to maintain an alkaline pH_i_, through unhindered NHE1 activity. It is worth noting, however, that NHE1 flux is normally measured experimentally under conditions in which oxygen tension is much greater than physiological levels of the myocardium. The magnitude of fluxes generated under these supra-physiological conditions must therefore be considered with caution.

## Data Availability Statement

The mass spectrometry proteomics data have been deposited to the ProteomeXchange Consortium *via* the PRIDE partner repository with the dataset identifier PXD022853.

## Ethics Statement

The animal study was reviewed and approved by Home Office Regulations (Schedule I of A(SP)A 1986), approved by National and University Ethics Committees, Home Office Guidance on the Operation of the Animals (Scientific Procedures) Act of 1986 and the University of Oxford institutional guidelines, Home Office under the Project License 30/3182.

## Author Contributions

HK, MR, GŞ, and PS collected and analyzed data. YC performed dietary interventions to produce anemia in mice. PS wrote the paper. All authors contributed to the article and approved the submitted version.

## Conflict of Interest

YC has received personal support from Vifor Pharma for work on iron biology not related to the current study. The remaining authors declare that the research was conducted in the absence of any commercial or financial relationships that could be construed as a potential conflict of interest.

## Supplementary Material

Supplementary Figure 1 Buffering capacity. Cells were first equilibrated with 30 mM NH_4_Cl, and then the superfusate was sequentially switched to 15, 10, 5, and 0 NH_4_Cl. Buffering capacity was calculated by relating the size of the pH_i_ drop with the change in intracellular [${\text{NH}}_{4}^{+}$], predicted from pH_i_ and the composition of the extracellular milieu. No difference between normoxic (*n* = 10 cells) and anoxic (*n* = 6 cells) conditions (from 3 rats).

Supplementary Figure 2 Effect of anoxia on fluorescence. **(A)** Rat myocyte loaded with cSNARF1 and superfused with calibration solutions containing 145 mM K^+^ and 10 μM nigericin, and titrated to pH 7.11 or 6.5. The switch between these solutions manipulates intracellular pH, and hence cSNARF1 fluorescence. A period of anoxia, however, produces a small artifact in the fluorescence ratio, particularly at low ratio. **(B)** Experiment repeated on rat myocytes loaded with BCECF. Periods of anoxia did not affect fluorescence ratio, indicating that BCECF is more resilient to oxygen depletion.
