# Supplementary figures and images for: Cardiomyocyte Na+/H+ Exchanger-1 Activity Is Reduced in Hypoxia
Source: Front Cardiovasc Med. 2021 Jan 27;7:617038. doi: 10.3389/fcvm.2020.617038 (PMC7873356; doi:10.3389/fcvm.2020.617038)

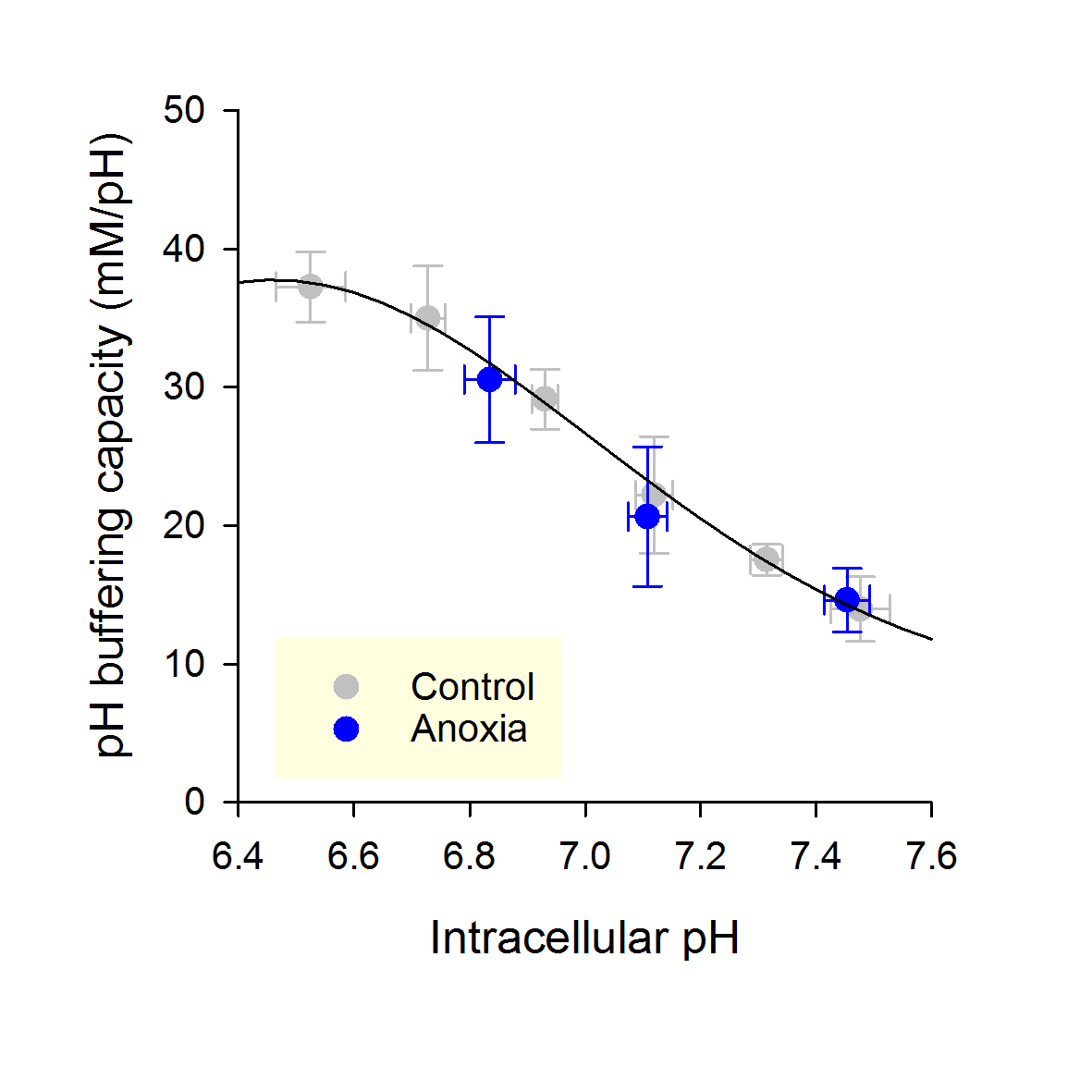

Supplement: Supplementary Figure 1 — Buffering capacity. Cells were first equilibrated with 30 mM NH4Cl, and then the superfusate was sequentially switched to 15, 10, 5, and 0 NH4Cl. Buffering capacity was calculated by relating the size of the pHi drop with the change in intracellular [NH4+], predicted from pHi and the composition of the extracellular milieu. No difference between normoxic (n = 10 cells) and anoxic (n = 6 cells) conditions (from 3 rats). [file Image_1.TIF]

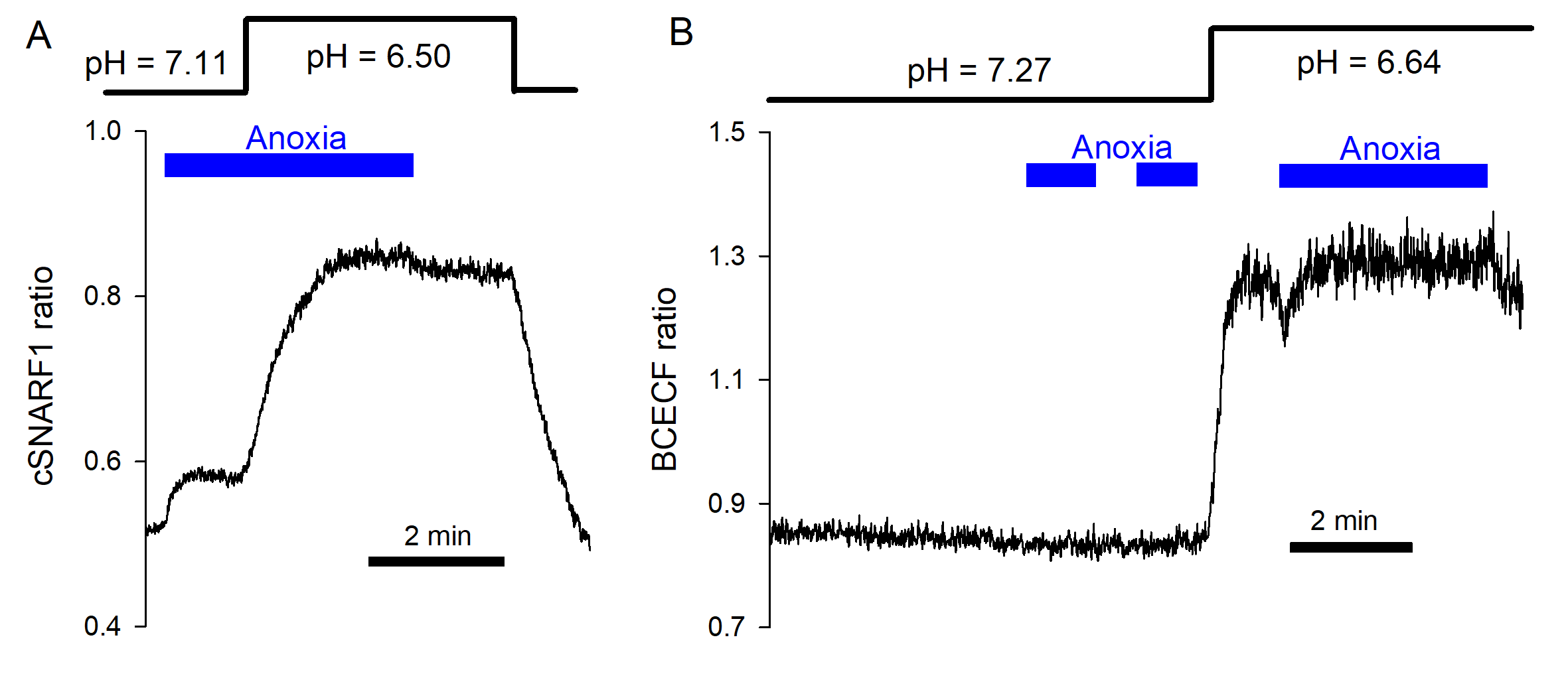

Supplement: Supplementary Figure 2 — Effect of anoxia on fluorescence. (A) Rat myocyte loaded with cSNARF1 and superfused with calibration solutions containing 145 mM K+ and 10 μM nigericin, and titrated to pH 7.11 or 6.5. The switch between these solutions manipulates intracellular pH, and hence cSNARF1 fluorescence. A period of anoxia, however, produces a small artifact in the fluorescence ratio, particularly at low ratio. (B) Experiment repeated on rat myocytes loaded with BCECF. Periods of anoxia did not affect fluorescence ratio, indicating that BCECF is more resilient to oxygen depletion. [file Image_2.TIF]
